# Single‐arm feasibility trial of manualized outpatient morita therapy for panic disorder with agoraphobia

**DOI:** 10.1002/pcn5.70430

**Published:** 2026-10-22

**Authors:** Kiwako Yokoyama, Daisuke Yamaichi, Shigetsugu Nakao, Mitsuhiro Nakamura, Hiroyuki Uchida, Hidehito Niimura

**Affiliations:** ^1^ Department of Neuropsychiatry Keio University School of Medicine Tokyo Japan; ^2^ Hamadayama Mental Clinic Tokyo Japan; ^3^ Shinano Mental Clinic Yokohama Kanagawa Japan; ^4^ Department of Clinical Psychology, Faculty of Clinical Psychology Taisho University Tokyo Japan

**Keywords:** agoraphobia, anxiety disorder, feasibility study, morita therapy, panic disorder

## Abstract

**Aim:**

This single‐arm feasibility trial evaluated the feasibility and safety of a manualized outpatient Morita therapy (MT) program for patients with panic disorder (PD) and agoraphobia in routine psychiatric care, and explored preliminary signals of symptom change.

**Methods:**

Ten outpatients with PD and agoraphobia were enrolled in a 12‐session manualized MT program. Feasibility outcomes included retention, attendance, and therapist adherence. Exploratory outcomes were the changes in scores of the Panic and Agoraphobia Scale (PAS), the Generalized Anxiety Disorder Scale‐7 (GAD‐7), the Patient Health Questionnaire‐9 (PHQ‐9), and the Short Form‐36 Health Survey (SF‐36), assessed from baseline to the 6‐month follow‐up. Data were analyzed using linear mixed‐effects models.

**Results:**

Feasibility criteria were met. The completion rate was 90%, with a mean attendance of 11.6/12 sessions. Therapists reported high manual fidelity and usability. Exploratory analyses showed improvements in PAS (Hedges' *g* = 1.53, 95% confidence interval [CI] 0.57–2.45) at 6‐month follow‐up, GAD‐7 (*g* = 1.24, 95% CI 0.38–2.06), PHQ‐9 (*g* = 1.05, 95% CI 0.25–1.80), and the Mental Component Summary of SF‐36 (*g* = 1.71, 95% CI 0.68–2.70). The Role/Social Component Summary of SF‐36 improved at post‐treatment (*g* = 0.82, 95% CI 0.09–1.51) but not at follow‐up (*g* = 0.45, 95% CI −0.19–1.06). No significant changes were observed in the Physical Component Summary of SF‐36 (*g* = −0.14, 95% CI −0.72–0.46).

**Conclusion:**

Manualized outpatient MT appears feasible and safe for patients with PD and agoraphobia. Preliminary effects on symptoms warrant confirmation in randomized controlled trials.

## INTRODUCTION

Panic disorder (PD), marked by recurrent and unexpected panic attacks (PAs), affects 1.7%–5.2% of the population and is more prevalent among women and individuals with psychosocial adversity.^1^, ^2^ About 80% of patients have psychiatric comorbidities, mainly anxiety (63.1%) and mood disorders (53.7%).^2^ Agoraphobia, occurring in 1.3%–2.6% of individuals,^1^, ^3^ often coexists with PD and is associated with greater severity and chronicity.^4^, ^5^

PD leads to significant occupational and social impairment. Individuals with anxiety disorders, including PD, face elevated risks of long‐term sick leave (odds ratio [OR] = 1.84) and reduced work capacity (OR = 2.13),^6^ accounting for 11% of recent work‐loss days, more than major depression or social anxiety disorder.^3^ PD markedly impairs Quality of Life (QoL).^7^, ^8^ Healthcare use is increased, largely due to health anxiety and somatic comorbidities, resulting in a substantial economic burden.^9^, ^10^ Moreover, PD is associated with elevated mortality (mortality rate ratio = 1.83).^11^

Although both pharmacological and psychological treatments are available for PD, evidence gaps remain, especially for patients with comorbid agoraphobia. Most randomized controlled trials (RCTs) include such patients, yet subgroup‐specific outcomes are rarely reported, impeding evaluation of tailored treatment efficacy.^12^ Selective serotonin reuptake inhibitors (SSRIs) and serotonin‐norepinephrine reuptake inhibitors (SNRIs) offer small‐to‐moderate benefits (standardized mean difference = −0.30), comparable to cognitive behavioral therapy (CBT; Hedges' *g* = 0.39).^12^, ^13^ However, CBT's long‐term efficacy is uncertain, with attenuated effects at 6–12 months and no significant advantage over active controls such as applied relaxation.^14^ Relapse is frequent, and booster sessions are often necessary^13^. Treatment uptake remains low (65.2% pharmacotherapy; 24.2% psychotherapy).^15^

Given the limitations of existing treatments, Morita therapy (MT) has attracted interest as a complementary or alternative intervention for PD. Originating in 1919 as an inpatient psychotherapy for neurotic disorders, now encompassing PD, somatic symptom disorder, and obsessive‐compulsive disorder (OCD), MT has evolved into outpatient and group formats^16^. In contrast to CBT, which focuses on controlling anxiety and emphasizes cognitive restructuring and systematic exposure to feared stimuli, MT does not aim for symptom elimination. Instead, it encourages patients to accept anxiety as a natural experience and to redirect their attention outward and live purposefully.^17^ Although MT shares with ACT the emphasis on accepting internal experiences, it differs in its theoretical foundation: MT conceptualizes anxiety as an expression of the “Desire to Live” and identifies “Toraware,” or pathological preoccupation with symptoms, as the central pathology.^18^ Behavioral tasks in MT are not structured exposure exercises but rather opportunities for patients to practice living with anxiety in their daily lives in accordance with their natural desires, consistent with the principle of “Arugamama,” which promotes acceptance of reality without resistance through emotional openness and work therapy aimed at shifting attention outward.^16^, ^17^, ^18^, ^19^ Since the 2000s, clinical epidemiological studies have supported MT's safety and utility for social anxiety disorder, generalized anxiety disorder (GAD), and OCD,^17^, ^20^, ^21^, ^22^, ^23^, ^24^, ^25^, ^26^, ^27^, ^28^, ^29^ yet no randomized controlled trials have assessed its effectiveness in PD or agoraphobia.^30^ Regarding outpatient Morita therapy, practice guideline have been published by the Japanese Society for Morita Therapy.^31^ This guideline, developed through expert consensus, has high clinical significance as a compilation of clinical wisdom. However, it does not specify concrete interventions for each treatment session and therefore is difficult to use as a structured manual suitable for efficacy studies. We then developed a brief, manualized MT program for outpatients with comorbid PD and agoraphobia.^32^ This pilot study evaluated the program's feasibility, safety, and preliminary effectiveness.

## MATERIALS AND METHODS

### Aims

The primary aim of this study was to evaluate the feasibility of delivering a manualized MT program for patients with PD and agoraphobia in routine psychiatric outpatient care. The secondary aim was to explore preliminary changes in PD and agoraphobia symptoms following treatment.

### Design

We conducted a single‐arm feasibility trial to assess the implementation of MT and its safety in patients with comorbid PD and agoraphobia, as a preparatory step toward an RCT. Feasibility indicators were collected during both recruitment and intervention phases. Clinical outcomes, including symptoms of PD, agoraphobia, depression, anxiety, and QoL, were measured at four primary time points: assessment before the first treatment session (baseline), at the end of the sixth treatment session (post‐session 6), at the end of treatment (post‐treatment), and at the 6‐month follow‐up (6‐mo FU). Additionally, PD and agoraphobia symptoms were assessed at the end of the third and ninth treatment sessions (post‐session 3 and post‐session 9). We also examined potential interaction effects of concurrent psychotropic medication use (benzodiazepines and antidepressants [AD]) based on drug use recorded from two months prior to treatment initiation through 6 months post‐treatment.

### Participants and recruitment

Participants were psychiatric outpatients from one hospital or two affiliated clinics in Tokyo, Japan. After receiving a full explanation of the study, eligible patients providing written informed consent were enrolled.

Inclusion criteria were: (a) meeting the Diagnostic and Statistical Manual of Mental Disorders, Fifth Edition (DSM‐5) diagnostic criteria for both PD and agoraphobia; (b) aged 18–59 years; (c) ability to attend at least nine treatment sessions during the study period; and (d) capacity to understand the study and provide informed consent voluntarily. The upper age limit of 59 years was set because PD and agoraphobia typically have an onset in early to middle adulthood,^33^ whereas older adults often present with different clinical characteristics and comorbidities (e.g., cognitive impairment, physical illnesses) that could complicate the assessment of feasibility.^34^

Exclusion criteria were: (a) psychotic or manic episodes; recent substance use disorder; organic brain disease; antisocial personality disorder; or intellectual disability; (b) other psychiatric conditions requiring priority treatment; (c) suicide risk or self‐harm; (d) unstable medical/social factors; and (e) concurrent psychotherapies.

A total of 15 patients were approached across the three sites (university hospital: 4; clinic A: 7; clinic B: 4). At clinic B, four individuals who contacted the study team after viewing an advertisement declined participation before eligibility screening. At the university hospital, one patient did not meet the diagnostic criteria for agoraphobia and was excluded at eligibility screening. Ultimately, 10 eligible patients were identified (university hospital: 3; clinic A: 4; clinic B: 3), and all provided informed consent and were enrolled.

One patient discontinued after 8 sessions due to early improvement (baseline PAS = 19; post‐session 6 PAS = 5) and, according to the predefined criterion of attending fewer than nine sessions, was considered a dropout. However, valid outcome data were obtained at both post‐treatment and 6‐mo FU, and this case was included in the analyses. Another patient completed all 12 sessions, but both the post‐treatment and 6‐mo FU assessments were conducted at inappropriate time points and were therefore treated as missing in the analyses. See Figure 1 for participant flow.

**Figure 1:**
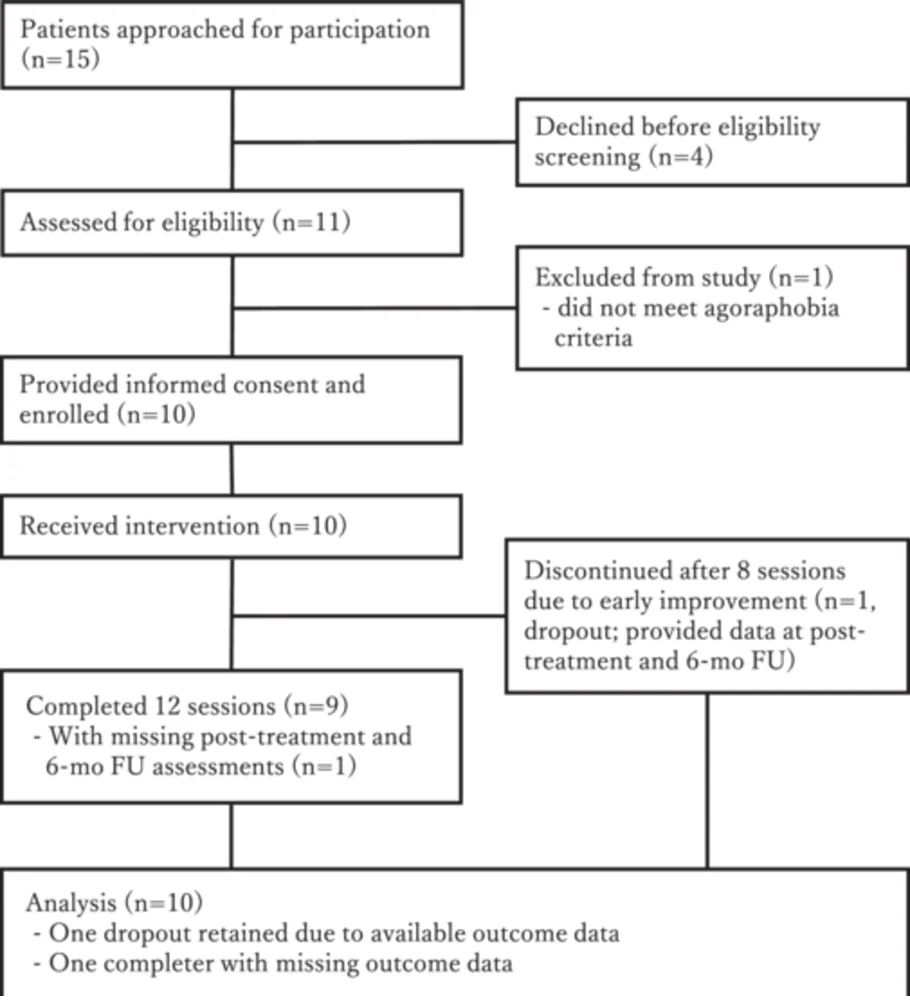
Flow of participants through the single‐arm feasibility trial.

### Treatment

The intervention comprised 12 individual face‐to‐face sessions (first up to 60 min, others up to 30 min) delivered at 1–3 week intervals.

### Therapeutic manual

We developed a structured manualized outpatient MT treatment manual for PD and agoraphobia. Based on the 10‐session structured outpatient MT proposed by Mitsuhiro Ichikawa,^35^ which to our knowledge provides the most systematically organized account of practice with interventions documented for each session, the intervention was extended to 12 sessions, and the manual was designed to allow the treatment process to be reviewed step‐by‐step. The treatment was divided into three stages. In the early stage (sessions 1–3), therapists conducted assessments, introduced the treatment, and provided psychoeducation on anxiety using slides. They identified patients' vicious symptom‐anxiety cycles and underlying “Desire to Live,” assigning behavioral tasks to help patients naturally experience anxiety without resistance, in line with the “Law of Emotion.” The middle stage (sessions 4–9) addressed not only symptom‐related themes but also interpersonal relationships and developmental history. The late stage (sessions 10–12) focused on each patient's way of life, the principle of “Arugamama,” and therapy termination.

If the treatment course proceeded favorably, patients would become capable of living with their anxiety. One homework task was assigned at each session, but patients were not required to submit written reports. For further details regarding the treatment content, please refer to Tables SA and SB.^36^, ^37^

### Therapists

The intervention was delivered by four psychiatrists, each with more than five years of clinical experience in MT. All therapists attended certified seminars (≥ 1 year lectures, ≥2 years supervised case discussions; 2 h × 10 annually).

Treatment fidelity was monitored by therapists documenting whether manual‐defined components (e.g., introduction of psychoeducation, assignment of homework, use of specific techniques) were delivered, with any deviations explicitly noted. In group supervision, each therapist presented cases, and adherence was reviewed by peers and HN, an MT‐certified physician, coauthor, and expert in the method. Points of adherence and areas for improvement were recorded during supervision and fed back into subsequent sessions.

### Outcome measures

#### Primary outcomes

##### Feasibility

Patient‐related feasibility was assessed using three criteria: (1) at least 50% of eligible patients consented to participate, (2) the mean attendance was at least 70% of the 12 scheduled sessions (≥ 8.4 sessions), and (3) the dropout rate (defined as attending fewer than 9 sessions) was less than 20%. Therapist‐related feasibility was evaluated post‐treatment based on responses to four items: clarity, usability, reliability of the manual, and willingness to continue its use beyond the study.

##### Safety

Adverse events (AEs) and serious adverse events (SAEs) were monitored throughout the study. Psychiatrists certified in Morita therapy evaluated patients at each session, and all new or worsening symptoms were documented as AEs, regardless of causality. Meetings were held at least once per month for safety evaluation and management, with additional meetings convened if necessary. An AE was defined as a temporary or minor impairment or an event requiring extended outpatient treatment. An SAE was defined as an event leading to death, life‐threatening conditions, permanent or significant impairment, or hospitalization. All AEs and SAEs were recorded, reported to the institutional head, and followed clinically until resolution or stabilization.

### Exploratory outcomes

#### Symptoms of panic disorder and agoraphobia, anxiety, and depression

Symptoms were assessed using the self‐rated version of the Panic and Agoraphobia Scale (PAS),^38^, ^39^ the Generalized Anxiety Disorder Scale‐7 (GAD‐7),^40^, ^41^, ^42^ and the Patient Health Questionnaire‐9 (PHQ‐9).^43^, ^44^ For all scales, higher scores indicate more severe symptoms.

#### Quality of life

Health‐related QoL was measured using the Japanese version of the 36‐Item Short Form Health Survey (SF‐36), which includes the Physical (PCS of SF‐36), Mental (MCS of SF‐36), and Role/Social (RCS of SF‐36) Component Summary scores. Higher scores reflect better health‐related QoL.^45^, ^46^, ^47^

### Data handling and statistical analysis

We used repeated‐measures linear mixed‐effects models (LMMs) to assess time‐related changes, with Time (and its interaction with medication group, when applicable) specified as fixed effects and a random intercept for participant (ID). Models assumed an independent residual error structure and were estimated using restricted maximum likelihood (REML). Degrees of freedom and p‐values were calculated using the Kenward–Roger method. Effect sizes were expressed as Hedges' g with 95% confidence intervals (CIs). Model fit was evaluated using marginal *R* ^2^ (*R* ^2^_m_; fixed effects only) and conditional *R* ^2^ (*R* ^2^_c_; fixed and random effects).^48^ Missing data were handled using available‐case analysis within the LMM framework, without multiple imputation. Pairwise comparisons between baseline and each follow‐up were conducted with the emmeans package (v1.10.0), applying false discovery rate (FDR) correction for multiple testing. In exploratory analyses, medication group (No Med [no medication], Bz [benzodiazepine only], AD+Bz [antidepressant + benzodiazepine]) was included as a between‐subject factor to examine Time × Group interactions.

All analyses were performed in R (RStudio v2025.05.0 + 496).

### Ethical considerations

This study was conducted in accordance with the Declaration of Helsinki and was approved by the Ethics Committee of Keio University School of Medicine (approval number: 20251242). This trial was registered in the University Hospital Medical Information Network (UMIN; trial ID: UMIN000042870; URL: https://center6.umin.ac.jp/cgi-open-bin/ctr/ctr_view.cgi?recptno = R000048932) on January 6, 2021, prior to participant enrollment (prospectively registered). All participants provided written informed consent prior to enrollment. No financial compensation or reimbursement was provided to the participants.

## RESULTS

Between December 2021 and January 2024, 10 patients were enrolled in the study, all of whom completed the full course of MT. Figure 1 illustrates patient flow, treatment adherence, and questionnaire response rates. One participant missed appropriately timed evaluations at post‐treatment and 6‐mo FU.

As shown in Table 1, participants were predominantly female and had longstanding agoraphobia symptoms. At baseline, participants had a mean PAS score of 23.0 (standard deviation [SD] = 5.8). Some of them had been on psychotropic medications, including AD and benzodiazepines, prior to treatment.

**Table 1:** Baseline characteristics.

		*n* or mean ± SD	%
Race	Japanese	10	100
Gender	Male	2	20
	Female	8	80
Age (years)		39.2 ± 13.4	
	18–29	3	30
	30–39	2	20
	40–49	1	10
	50–59	4	40
Education (years)		14.8 ± 2.1	
Employment Status	Employed (full‐time)	2	20
	On leave from full‐time employment	2	20
	Student	2	20
	Unemployed	3	30
	Homemaker	1	10
Marital Status	Married	6	60
	Single, widowed, or divorced	4	40
Age of onset (years old)		34.7 ± 16.1	
Duration of the agoraphobia (years)		4.5 ± 7.9	
PAS (score)		23.0 ± 5.8	
Coexisting psychiatric disorder	Panic disorder	10	100
	Major depressive disorder	2	20
	Obsessive‐Compulsive Disorder	2	20
	Adjustment disorder	1	10
	Social Anxiety Disorder	2	20
	Somatic Symptom Disorder	1	10
Coexisting physical disorder	Behcet's syndrome	1	10
History of abuse		4	40
Baseline psychotropic medication	SSRI/SNRI/other antidepressants	2	20
	Benzodiazepines	3	30
Recent change in antidepressant (within 2 months)	Yes	1	10
	No	9	90

*Note*: Values are presented as number (%) or mean ± SD.

Abbreviations: PAS, Panic and Agoraphobia Scale; SD, standard deviation; SNRI, serotonin–noradrenaline reuptake inhibitor; SSRI, selective serotonin reuptake inhibitor.

### Feasibility and safety

100% of eligible patients consented to participate. Participants attended a mean of 11.6 out of 12 sessions (SD = 1.26, median = 12, interquartile range [IQR] = 0, range = 8–12). One participant discontinued after 8 sessions due to early improvement and was classified as a dropout according to the predefined criterion (< 9 sessions), resulting in a dropout rate of 10%. All other participants attended all 12 sessions. No intervention‐related AEs or SAEs were observed. The patient who discontinued due to early improvement, and all other participants completed treatment or follow‐up without safety concerns.

All therapists rated the treatment manual highly in terms of clarity, usability, reliability, and willingness for continued use. Feasibility outcomes are summarized in Table 2.

**Table 2:** Feasibility criteria and outcomes.

	Patient‐related feasibility criteria	Outcome
1	At least 50% of eligible patients consented to participate.	Yes. 100% consented.
2	The mean attendance was at least 70% of the 12 scheduled sessions (≥8.4 sessions)	Yes. It was 11.6 of 12 sessions.
3	The dropout rate (defined as attending fewer than 9 sessions) was less than 20%.	Yes. It was 10%.
	Therapist‐related feasibility criteria	Outcome
1	The manual is easy to understand.	Yes. All therapists agreed.
2	The manual is easy to use.	Yes. All therapists agreed.
3	The manual is reliable.	Yes. All therapists agreed.
4	I would like to continue using the manual even after the study has concluded.	Yes. All therapists agreed.

### Changes in each scale from baseline

Figure 2 displays estimated means over time for all scales, with asterisks denoting significant differences from baseline (FDR‐adjusted p‐values; see Table 3). Most scales showed significant improvement during the intervention, except for the PCS of SF‐36, which did not change significantly, and the RCS of SF‐36, which improved only at post‐treatment but not at 6‐mo FU. Pairwise comparisons with Hedges' *g* are shown in Table 4, and *R* ^2^ values from the LMM analyses are summarized in Table SC. These analyses were exploratory and should be interpreted with caution.

**Figure 2:**
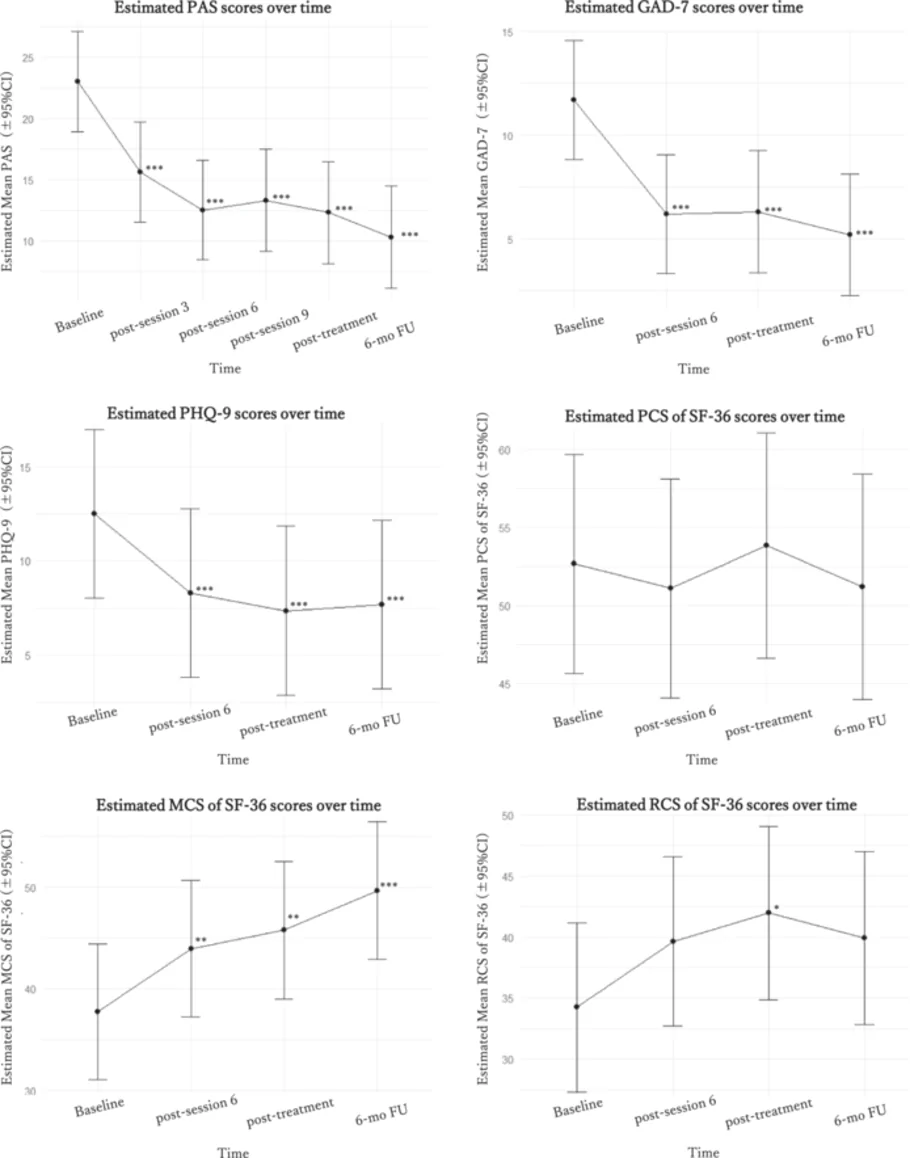
Estimated means at each time point for all scales (LMM). *Note*: Error bars show 95% CIs (unadjusted). Asterisks reflect FDR‐adjusted p values from baseline versus timepoint contrasts (Kenward–Roger *df*). Analyses were based on available cases at each time point; no imputation was performed. Analyses were exploratory. *p < 0.05,**p < 0.01,***p < 0.001. 6‐mo FU, the 6‐month follow‐up; baseline, assessment before the first treatment session; CI, confidence interval; GAD‐7, Generalized Anxiety Disorder Scale‐7; LMM, linear mixed‐effects model; MCS of SF‐36, Mental Component Summary of the SF‐36; PAS, Panic and Agoraphobia Scale; PCS of SF‐36, Physical Component Summary of the SF‐36; PHQ‐9, Patient Health Questionnaire‐9; post‐session 3, the end of the third treatment session; post‐session 6, the end of the sixth treatment session; post‐session 9, the end of the ninth treatment session; post‐treatment, the end of treatment; RCS of SF‐36, Role/Social Component Summary of the SF‐36; SF‐36, 36‐Item Short Form Health Survey.

**Table 3:** Fixed effects from the linear mixed‐effects model.

Scale	Term	Estimated difference from baseline	95% CI	SE	df	*t*	*p*
PAS	Intercept (baseline)	23.00	18.92–27.08	1.96	20.43	11.74	< 0.001
	Time: post‐session 3	−7.40	−11.12 to −3.68	1.84	41.83	−4.02	< 0.001
	Time: post‐session 6	−10.50	−14.22 to −6.78	1.84	41.83	−5.70	< 0.001
	Time: post‐session 9	−9.70	−13.54 to −5.87	1.90	41.95	−5.11	< 0.001
	Time: post‐treatment	−10.72	−14.56 to −6.88	1.90	42.01	−5.63	< 0.001
	Time: 6‐mo FU	−12.72	−16.56 to −8.88	1.90	42.01	−6.69	< 0.001
GAD‐7	Intercept (baseline)	11.70	8.84–14.56	1.36	18.20	8.59	< 0.001
	Time: post‐session 6	−5.50	−8.13 to −2.87	1.28	25.14	−4.30	< 0.001
	Time: post‐treatment	−5.39	−8.12 to −2.65	1.33	25.46	−4.06	< 0.001
	Time: 6‐mo FU	−6.50	−9.23 to −3.76	1.33	25.46	−4.90	< 0.001
PHQ‐9	Intercept (baseline)	12.50	8.03–16.97	2.02	10.38	6.20	< 0.001
	Time: post‐session 6	−4.20	−6.06 to −2.34	0.91	24.94	−4.64	< 0.001
	Time: post‐treatment	−5.15	−7.09 to −3.21	0.94	25.01	−5.47	< 0.001
	Time: 6‐mo FU	−4.82	−6.76 to −2.88	0.94	25.01	−5.12	< 0.001
PCS of SF‐36	Intercept (baseline)	52.68	45.61–59.75	3.33	15.76	15.83	< 0.001
	Time: post‐session 6	−1.58	−7.97–4.81	3.10	23.30	−0.51	0.62
	Time: post‐treatment	1.16	−5.47–7.79	3.22	23.64	0.36	0.72
	Time: 6‐mo FU	−1.49	−8.12–5.15	3.22	23.64	−0.46	0.65
MCS of SF‐36	Intercept (baseline)	37.75	31.07–44.43	3.09	13.00	12.2	< 0.001
	Time: post‐session 6	6.20	1.83–10.57	2.12	25.10	2.92	0.007
	Time: post‐treatment	8.01	3.47–12.55	2.21	25.26	3.63	0.001
	Time: 6‐mo FU	11.90	7.36–16.44	2.21	25.26	5.40	< 0.001
RCS of SF‐36	Intercept (baseline)	34.23	27.68–40.79	3.26	15.47	10.49	< 0.001
	Time: post‐session 6	5.41	−0.14–10.96	2.70	25.14	2.01	0.06
	Time: post‐treatment	7.73	1.96–13.50	2.80	25.38	2.76	0.01
	Time: 6‐mo FU	5.67	−0.10–11.44	2.80	25.38	2.03	0.054

*Note*: Estimates represent fixed effects from a linear mixed‐effects model, with baseline as the reference. 95% CIs were estimated using emmeans() with Kenward–Roger degrees of freedom adjustment. For PAS, GAD‐7, and PHQ‐9, negative values indicate symptom improvement; for SF‐36 component scores, positive values indicate improvement. All available cases were analyzed; no missing data were imputed. P‐values were adjusted for multiple comparisons using the false discovery rate (FDR) method. Analyses were exploratory.

Abbreviations: 6‐mo FU, the 6‐month follow‐up; baseline, assessment before the first treatment session; CI, confidence intervals; df, degrees of freedom; GAD‐7, Generalized Anxiety Disorder Scale‐7; MCS of SF‐36, Mental Component Summary of the SF‐36; PAS, Panic and Agoraphobia Scale; PCS of SF‐36, Physical Component Summary of the SF‐36; PHQ‐9, Patient Health Questionnaire‐9; post‐session 3, the end of the third treatment session; post‐session 6, the end of the sixth treatment session; post‐session 9, the end of the ninth treatment session; post‐treatment, the end of treatment; RCS of SF‐36, Role/Social Component Summary of the SF‐36; SE, standard error; SF‐36, 36‐Item Short Form Health Survey.

**Table 4:** Pairwise comparisons of each score and effect sizes (Hedges' *g*).

Scale	Comparison	MD	95% CI	*p*	Hedges' *g*	95% CI for Hedges' *g*
PAS	Baseline vs. post‐session 3	−7.40	−11.12 to −3.68	< 0.001	1.11	0.33–1.85
	Baseline vs. post‐session 6	−10.50	−14.22 to −6.78	< 0.001	1.54	0.62–2.43
	Baseline vs. post‐session 9	−9.70	−13.54 to −5.87	< 0.001	1.06	0.26–1.83
	Baseline vs. post‐treatment	−10.72	−14.56 to −6.88	< 0.001	1.13	0.31–1.92
	Baseline vs. 6‐mo FU	−12.72	−16.56 to −8.88	< 0.001	1.53	0.57–2.45
GAD‐7	Baseline vs. post‐session 6	−5.50	−8.13 to −2.87	< 0.001	1.32	0.48–2.13
	Baseline vs. post‐treatment	−5.39	−8.12 to −2.65	< 0.001	1.00	0.22–1.75
	Baseline vs. 6‐mo FU	−6.50	−9.23 to −3.76	< 0.001	1.24	0.38–2.06
PHQ‐9	Baseline vs. post‐session 6	−4.20	−6.06 to −2.34	< 0.001	1.60	0.66–2.51
	Baseline vs. post‐treatment	−5.15	−7.09 to −3.21	< 0.001	1.62	0.63–2.58
	Baseline vs. 6‐mo FU	−4.82	−6.76 to −2.88	< 0.001	1.05	0.25–1.80
PCS of SF‐36	Baseline vs. post‐session 6	−1.58	−7.97–4.81	0.72	−0.14	−0.71–0.43
	Baseline vs. post‐treatment	1.16	−5.47–7.79	0.72	0.07	−0.52–0.66
	Baseline vs. 6‐mo FU	−1.49	−8.12–5.15	0.72	−0.14	−0.72–0.46
MCS of SF‐36	Baseline vs. post‐session 6	6.20	1.83–10.57	0.007	1.22	0.41–2.00
	Baseline vs. post‐treatment	8.01	3.47–12.55	0.001	1.00	0.22–1.74
	Baseline vs. 6‐mo FU	11.90	7.36–16.44	< 0.001	1.71	0.68–2.70
RCS of SF‐36	Baseline vs. post‐session 6	5.41	−0.14–10.96	0.056	0.56	−0.07–1.17
	Baseline vs. post‐treatment	7.73	1.96–13.50	0.032	0.82	0.09–1.51
	Baseline vs. 6‐mo FU	5.67	−0.10–11.44	0.056	0.45	−0.19–1.06

*Note*: Values are based on pairwise comparisons from linear mixed‐effects models with Baseline as the reference. MD are shown with 95% CIs. Hedges' *g* values were calculated for paired comparisons and oriented so that positive values indicate symptom improvement. *p*‐values were adjusted for multiple comparisons using FDR method with Kenward–Roger degrees of freedom correction. Missing data were handled under the assumption of missing at random using mixed‐effects modeling without imputation. Analyses were exploratory.

Abbreviations: 6‐mo FU, the 6‐month follow‐up; baseline, assessment before the first treatment session; CI, confidence intervals; FDR, false discovery rate; GAD‐7, Generalized Anxiety Disorder Scale‐7; MCS of SF‐36, Mental Component Summary of the SF‐36; MD, mean difference; PAS, Panic and Agoraphobia Scale; PCS of SF‐36, Physical Component Summary of the SF‐36; PHQ‐9, Patient Health Questionnaire‐9; post‐session 3, the end of the third treatment session; post‐session 6, the end of the sixth treatment session; post‐session 9, the end of the ninth treatment session; post‐treatment, the end of treatment; RCS of SF‐36, Role/Social Component Summary of the SF‐36; SF‐36, 36‐Item Short Form Health Survey.

### Panic and Agoraphobia Scale (PAS)

The PAS scores improved significantly from baseline (23.0, 95% CI 18.9–27.1) to post‐session 3 (15.6, 95% CI 11.5–19.7), post‐session 6 (12.5, 95% CI 8.4–16.6), post‐session 9 (13.3, 95% CI 9.1–17.5), and post‐treatment (12.3, 95% CI 8.1–16.5), with sustained improvement at 6‐mo FU (10.3, 95% CI 6.1–14.5). For the primary comparison (baseline vs. 6‐mo FU), the effect size was large (Hedges' *g* = 1.53, 95% CI 0.57–2.45, *p* < 0.001; *R* ^2^m = 0.32, *R* ^2^c = 0.70).

### Generalized Anxiety Disorder‐7 (GAD‐7)

GAD‐7 scores significantly decreased from baseline (11.7, 95% CI 8.8–14.6) to post‐treatment (6.3, 95% CI 3.4–9.3), with further improvement maintained at 6‐mo FU (5.2, 95% CI 2.3–8.2). For the primary comparison (baseline vs. 6‐mo FU), the effect size was large (Hedges' *g* = 1.24, 95% CI 0.38–2.06, *p* < 0.001; *R* ^2^m = 0.27, *R* ^2^c = 0.68).

### Patient Health Questionnaire‐9 (PHQ‐9)

PHQ‐9 scores significantly declined from baseline (12.5, 95% CI 8.0–17.0) to post‐treatment (7.4, 95% CI 2.9–11.8). Although scores slightly worsened at 6‐mo FU (7.7, 95% CI 3.2–12.2), the improvement from baseline remained statistically significant. For the primary comparison (baseline vs. 6‐mo FU), the effect size was large (Hedges' *g* = 1.05, 95% CI 0.25–1.80, *p* < 0.001; *R* ^2^m = 0.10, *R* ^2^c = 0.91).

### Short Form‐36 (SF‐36)

Physical health component summary (PCS of SF‐36): No statistically significant changes were observed from baseline (52.7, 95% CI 45.7–59.7) to post‐treatment (53.8, 95% CI 46.6–61.0) and 6‐mo FU (51.2, 95% CI 44.0–58.4). The effect size for the primary comparison (baseline vs. 6‐mo FU) was negligible (Hedges' *g* = ‐0.14, 95% CI −0.72–0.46, *p* = 0.65; *R* ^2^m = 0.01, *R* ^2^c = 0.57), consistent with the absence of significant change.

Mental health component summary (MCS of SF‐36): Significant improvement was observed from baseline (37.8, 95% CI 31.1–44.4) to post‐treatment (45.8, 95% CI 39.0–52.5) and further sustained at 6‐mo FU (49.7, 95% CI 42.9–56.4). For the primary comparison (baseline vs. 6‐mo FU), the effect size was large (Hedges' *g* = 1.71, 95% CI 0.68–2.70, *p* < 0.001; *R* ^2^m = 0.17, *R* ^2^c = 0.80).

Role/social functioning component summary (RCS of SF‐36): The scores significantly improved from baseline (34.2, 95% CI 27.3–41.2) to post‐treatment (42.0, 95% CI 34.9–49.0, *p* = 0.01). At 6‐mo FU, scores remained higher than baseline, although the difference did not reach statistical significance (39.9, 95% CI 32.8–47.0, *p* = 0.054). For the primary comparison (baseline vs. 6‐mo FU), the effect size was medium (Hedges' *g* = 0.45, 95% CI −0.19–1.06, *p* = 0.054; *R* ^2^m = 0.07, *R* ^2^c = 0.68).

As an exploratory analysis, we examined responder rates for the SF‐36 component summary scores. The minimal clinically important difference (MCID, also referred to as the minimally important difference [MID]) was defined as 0.5 SD at baseline, based on a distribution‐based approach.^49^ The proportions of participants at each timepoint who achieved improvements greater than or equal to the MCID are presented in Table SD.

In a separate exploratory analysis, we also calculated the proportions of participants whose SF‐36 component scores reached the norm‐based standard of 50.0 (±10.0), derived from the US general population^50^ and adapted to Japanese population data.^47^ These proportions at each timepoint are summarized in Table SE.

### Medication interaction with PAS

As an exploratory analysis, no significant interaction effects of time × medication group (No Med [*n* = 5], Bz [*n* = 3], AD + Bz [*n* = 2]) on PAS scores were observed during the period from baseline to 6‐mo FU (all *p* > 0.20; *R* ^2^m = 0.39, *R* ^2^c = 0.71) (Tables SF and SG).

## DISCUSSION

Our manualized outpatient MT was safe and well‐tolerated, with no AEs and SAEs. The 12‐session program with 6‐mo FU demonstrated feasibility and acceptability, with exploratory analyses suggesting sustained symptom reduction on the PAS.

Unlike CBT, which emphasizes cognitive restructuring and exposure‐based techniques, MT operates from a fundamentally different paradigm, encouraging patients to shift their attention away from symptom control and toward purposeful living through structured activity and acceptance of internal experiences. This distinct approach may benefit patients reluctant to engage in CBT or pharmacotherapy.

This study assessed outcomes on the PHQ‐9, GAD‐7, and SF‐36. Exploratory analyses indicated improvements in depression, generalized anxiety, and mental functioning. There was also a trend toward improvement in the RCS of SF‐36, although it did not consistently reach statistical significance. This may reflect that improvements in role/social functioning require longer behavioral expansion, with daily activities and interactions gradually strengthening social roles. By contrast, the PCS of SF‐36 showed no significant change, which aligns with meta‐analytic evidence indicating that PCS is generally less responsive to anxiety treatments because it reflects objective physical limitations rather than subjective distress.^51^

Although the interaction between medication group and time was not statistically significant (*p* > 0.2), these findings are preliminary, and the potential influence of pharmacotherapy remains inconclusive and should be interpreted with caution, given the very small and uneven sample sizes (No Med = 5, Bz = 3, AD + Bz = 2).

This study has notable limitations. The small, uncontrolled design limits causal inference and prevents firm conclusions regarding efficacy. Selection bias is possible, and treatment was limited to Japanese patients with MT in a single cultural context, restricting generalizability, and cross‐cultural validation is needed.

Despite these limitations, this pilot study provides preliminary indications that MT may be feasible, safe, and acceptable for patients with PD and agoraphobia. Future controlled trials are required to clarify the clinical effects and comparative utility of MT in diverse settings.

## CONCLUSION

This pilot study suggests that manualized outpatient MT is feasible and safe for patients with PD and agoraphobia. High adherence and the absence of safety concerns support its potential acceptability. However, given the small sample size and lack of a control group, the observed symptom changes should be interpreted as exploratory, and further RCTs are needed to evaluate efficacy and generalizability.

## AUTHOR CONTRIBUTIONS

Kiwako Yokoyama, Daisuke Yamaichi, Shigetsugu Nakao, and Hidehito Niimura contributed to the study design, intervention design, protocol development, data collection, and clinical interventions. Mitsuhiro Nakamura contributed to the intervention design. Hidehito Niimura supervised the trial. Kiwako Yokoyama performed the statistical analysis. Kiwako Yokoyama and Daisuke Yamaichi drafted the manuscript. Shigetsugu Nakao, Mitsuhiro Nakamura, Hiroyuki Uchida, and Hidehito Niimura critically revised the manuscript for important intellectual content. All authors read and approved the final manuscript.

## CONFLICT OF INTEREST STATEMENT

H.N. is an Editorial Board Member of PCN Reports and was not involved in the editorial review or the decision to accept this article. The remaining authors declare no conflicts of interest.

## ETHICS APPROVAL STATEMENT

This study was conducted in accordance with the Declaration of Helsinki and was approved by the Ethics Committee of Keio University School of Medicine (approval number: 20251242). This trial was registered in the University Hospital Medical Information Network (UMIN; trial ID: UMIN000042870; URL: https://center6.umin.ac.jp/cgi-open-bin/ctr/ctr_view.cgi?recptno=R000048932) on January 6, 2021, prior to participant enrollment (prospectively registered). All participants provided written informed consent prior to enrollment. No financial compensation or reimbursement was provided to the participants.

## PATIENT CONSENT STATEMENT

N/A.

## CLINICAL TRIAL REGISTRATION

N/A.

## Supporting information

Supporting File 1.

Supporting File 2.

Supporting File 3.

Supporting File 4.

Supporting File 5.

Supporting File 6.

Supporting File 7.

Supporting File 8.

## Data Availability

The data sets generated and/or analyzed during the current study are not publicly available due to patient privacy and ethical restrictions but are available from the corresponding author on reasonable request.
